# Physical and psychological long-term outcome after traumatic brain injury in children and adult patients

**DOI:** 10.1186/1477-7525-12-26

**Published:** 2014-02-26

**Authors:** Hagen Andruszkow, Ezin Deniz, Julia Urner, Christian Probst, Orna Grün, Ralf Lohse, Michael Frink, Christian Krettek, Christian Zeckey, Frank Hildebrand

**Affiliations:** 1 Department of Orthopaedic Trauma, Aachen University, Pauwelsstraße 30, 52074 Aachen, Germany; 2 Department of Trauma and Orthopaedic Surgery, Cologne Merheim Medical Center, Faculty of Health-School of Medicine, Witten/Herdecke University, Ostmerheimer Straße 200, 51109 Cologne, Germany; 3 Hannover Re Insurance, Karl-Wiechert-Allee 50, 30625 Hannover, Germany; 4 Department for Trauma, Hand and Reconstructive Surgery, University Medical Center Marburg, Baldingerstr, 35043 Marburg, Germany; 5 Trauma Department, Hannover Medical School, Carl Neuberg-Str 1, 30625 Hannover, Germany

**Keywords:** Traumatic brain injury, Long-term outcome, Morbidity, Children

## Abstract

**Background:**

Several studies have indicated that younger age is associated with worse recovery after pediatric traumatic brain injury (TBI) compared to elder children. In order to verify this association between long-term outcome after moderate to severe TBI and patient’s age, direct comparison between different pediatric age groups as well as an adult population was performed.

**Methods:**

This investigation represents a retrospective cohort study at a level I trauma center including patients with moderate to severe, isolated TBI with a minimum follow-up of 10 years. According to their age at time of injury, patients were divided in pre-school (0–7 years), school (8–17 years) and adult (18–65 years) patients. Physical examination and standardized questionnaire on physical and psychological aspects (Glasgow Outcome Scale, Barthel Index, Impact of Event Scale, Hospital Anxiety and Depression Scale, short form 12) were performed.

**Results:**

135 traumatized patients were included. Physical and psychological long-term outcome was associated with injury severity but not with patients’ age at time of injury. Outcome recovery measured by Glasgow Outcome Scale was demonstrated with best results for pre-school aged children (p = 0.009). According to the Hospital Anxiety and Depression Scale an increased incidence of anxiety (p = 0.010) and depression (p = 0.026) was evaluated in older patients.

**Conclusion:**

Long-term outcome perceptions after moderate to severe TBI presented in this study question current views of deteriorated recovery for the immature brain. The sustained TBI impact seemed not to reduce the child’s ability to overcome the suffered impairment measured by questionnaire based psychological, physical and health related outcome scores. These results distinguish the relevance of rehabilitation and family support in the long term.

## Background

Traumatic Brain Injury (TBI) is known to represent a major public health concern potentially resulting in death or neurological impairment [1,2]. Especially children are at high risk to sustain TBI with an incidence of 345 in 100,000 children annually [3,4], and 1 of 30 newborns to suffer a TBI by the age of 16 years [5]. Due to an increasing clinical experience and improved treatment algorithms, overall mortality decreased during the last decades in traumatized children with TBI [5]. However, prediction of outcome in survivors is described to be complex, as several interacting factors like injury severity, rehabilitation and social support have been shown to have a significant influence on the incidence of residual impairments [6].

Similar to TBI in adult populations, the nature and severity of TBI is closely related to outcome after pediatric TBI [6,7]. However, anatomic variances to adults like a disproportional large and heavy head with weak neck muscles as well as greater flexibility of cranial bones minimize focal brain injuries but increase the risk of diffuse brain injuries [5,8-10]. Despite the common suggestion, that children’s brain are capable to adapt to the impact of considerable TBI impacts, several studies indicate that younger age is associated with worse recovery after injury compared to elder children [4,9,10]: In this context, it was concluded that young children might be more vulnerable to disruptions caused by TBI compared to elder children as their brain is more rapidly developing with considerable cognitive skill maturation [5,6,11,12]. Once this cerebral development is interrupted in the early stage, sustained deficits seem to reduce the child’s ability to acquire knowledge and skills to manage or minimize the impairment [9,10]. In contrast to this suggestion, other studies found that the association of injury severity and outcome deficits diminishes with increasing time since injury in children [6,10,13], suggesting other influencing factors like rehabilitation [14] and family environment being more important than the sustained injury [9,15].

In the current study we aimed to observe whether long-term outcome after moderate to severe TBI in pediatric patients is influenced by patients’ age. Furthermore, it was investigated whether pediatric patients have a better recovery after TBI compared to adults.

## Methods

This study followed the guidelines of the revised UN declaration of Helsinki in 1975 and its latest amendment in 1996 (42nd general meeting). The study was approved by the institutional ethical review board (No. 6221). Written informed consent was obtained from all adult participants. In case of children (aged <18 years), parental permission and child assent were used for participation. One or both parents accompanied the questioning and re-examination.

The study was approved by the institutional ethical review board at the Hannover Medical School, Hannover, Germany (No. 6221).

### Study design and population

The investigation was designed as a retrospective cohort study at a level I trauma center. The clinical database was generated for this study including all patients with TBI at least 10 years after trauma referring to December 1^st^ 2009 [16]. Participants have not been involved to other studies. Assessment and re-examination of the included patients was performed between December 1^st^ 2009 and October 31^st^ 2011. Patients were analyzed by our databank and included in the study if the following criteria were fulfilled:

– Isolated moderate to severe TBI classified by the Glasgow Coma Scale (GCS): moderate (GCS 9–12) and severe (GCS 3–8) TBI

– Minimum follow-up at least 10 years after trauma

The exclusion criteria were as follows:

– Additional severe injury defined as Abbreviated Injury Scale (AIS) > 2 points until follow-up

– Physical or mental handicap previous to TBI

The following study groups were designed:

“Pre-school group” included patients between 0 and 7 years of age at time of injury, “school group” between 8 and 17 years and the “adult group” included participants between 18 and 65 years.

### Contacting of patients and examination

Patients were recruited according to an established recruitment process [17]: Apriori, patients residences were gathered from hospital medical records. If patients had moved, up to three different registration offices were contacted by mail in order to determine the current address. Afterwards, the patients were contacted by mail in a letter describing the purposes of the present study and asked to make an appointment. The patients were contacted via mail and subsequently by phone up to three times. If none of these attempts was successful or three appointments were missed, patients were documented as “not available” for follow-up.

Patients with moderate and severe TBI were re-examined by an experienced orthopaedic trauma surgeon. For re-examination a previously described self-administered patient questionnaire and a standardized physical examination were used [18].

### Life changing events

All participants were asked for life changing events that might have influenced outcome subjectively.

### Traumatic brain injury

TBI was classified based on the initial Glasgow Coma Scale (GCS) [19] identifying patients with moderate (GCS 9–12) and severe (GCS 3–8) TBI [3,20,21]. Due to the fact that previous studies established to combine patients with moderate to severe TBI into one study group [20,22], patients with moderate and severe TBI were summarized into one group in the presented study as well to guarantee comparability [20,22].

### Demographic data and injury severity

Demographic and clinical data were extracted from patients’ charts including patients’ age and gender.

Injury severity was measured by the maximum Abbreviated Injury Scale (maximum AIS) and the Injury Severity Score (ISS) [23]. The 2005 updated version of the AIS was used.

Besides the aforementioned differentiation of TBI severity, an additional classification according to morphological criteria based on computed tomography (CT) was used. Therefore, the first acquired CT scan was evaluated independently and blinded by an experienced trauma surgeon (F.H.) based on the established Traumatic Coma Databank (TCDB) score by Marshall et al. [24]. This CT classification differentiates between mass lesions and permits a further discrimination of patients with diffuse injuries into 4 categories, taking into account signs of intracranial pressure (present or absent basal cisterns, midline shift) (Table 1) [24,25].

**Table 1:** TCDB classification of TBI severity

**Category**	**Definition**
Diffuse injury I (no visible pathology)	No visible intracranial pathology seen on CT scan
Diffuse injury II	Cisterns are present with midline shift of 0–5 mm and/or lesions densities present; no high or mixed density lesion >25 cm3 may include bone fragments and foreign bodies
Diffuse injury III (swelling)	Cisterns compressed or absent with midline shift of 0–5 mm; no high or mixed density lesion >25 mm
Diffuse injury IV (shift)	Midline shift >5 mm; no high or mixed density lesion >25 cm3
Evacuated mass lesion V	Any lesion surgically evacuated
Non-evacuated mass lesion VI	High or mixed density lesion >25 cm3; not surgically evacuated

### Outcome assessment

In order to assess the neurological outcome the *Glasgow outcome scale (GOS)* with the following description was used [26]:

– Persistent vegetative state: Patient exhibits no obvious cortical function.

– Severe Disability: (Conscious but disabled). Patient depends upon others for daily support due to mental or physical disability or both

– Moderate Disability. (Disabled but independent). Patient is independent as far as daily life is concerned. The disabilities found include varying degrees of dysphasia, hemiparesis, or ataxia, as well as intellectual and memory deficits and personality changes.

– Good Recovery. Resumption of normal activities even though there may be minor neurological or psychological deficits.

Furthermore, the presence of post-trauma mental health was observed. Therefore, the *short from 12 (SF-12)* was used for patient assessment as a modified version of the SF-36 in German language [27]. It implies *Physical Component Summary Scale (PCS)* and *Mental Component Summary Scale (MCS)*[28].

In order to evaluate physical outcome, the established *Barthel Index* was used. This 10-item assessment tool evaluates physical dependence in activities of daily living [29]. Two items regarding grooming and bathing were assessed using a 2-point scale (0 and 5 points); 6 items regarding feeding, toilet use, ascending and descending stairs, dressing, controlling bowels, and bladder control were scored on a 3-point scale (0, 5, and 10 points); and 2 items regarding moving from a wheelchair to bed and returning and walking on a level surface were evaluated on a 4-point scale (0, 5, 10, and 15 points) [30]. Total possible scores range from 0 to 100, with lower scores representing greater dependency [30]. It has been reliably employed in settings focusing outcome after TBI [31].

The *Hospital Anxiety and Depression Scale (HADS)* represents a questionnaire designed to detect anxiety and depression [32]. Its items are rated on a four-point scale ranging from absence of symptoms to maximum symptomatology [33]. The clinical significance of anxiety and depression were calculated on a scale whereby scores of 0 to 7 are non-cases, 8 to 10 are borderline cases and scores of 11 to 21 indicate patients whose conditions represent psychiatric assessment (cases) [33]. The validity of this score has previously been demonstrated in studies focusing on pediatric TBI [33,34].

The *Impact of Event Scale (IES)*[35] was evaluated to assess the psychological stress reactions following TBI. It consists of a validated 15-item self-report scale that assesses two post traumatic stress disorder (PTSD) symptom-cluster: intrusion (7 items) and avoidance (8 items) symptoms [35,36]. IES intrusion scores range from 0 to 35 points while avoidance scores range from 0 to 40 points [37]. The summarized mean IES score has been revealed to identify patients with PTSD [37].

### Statistical analysis

The data were analyzed using the Statistical Package for the Social Sciences (SPSS; version 22; IBM Inc., Somers, NY, USA). Incidences are presented with counts or percentages while continuous values are presented as mean ± standard deviation (SD). Differences between the groups were evaluated with analysis of variance (ANOVA) for continuous data, while Pearson’s χ^2^-test was used for categorical values. The Tukey post-hoc test was used when appropriate to identify differences between the aforementioned classified study groups. The Pearson correlation and Spearman rank correlation coefficients were performed to determine the association between age at time of injury, injury severity and the miscellaneous long-term outcome parameters. A two sided p-value < 0.05 was considered to be significant.

## Results

### Demographic data

Overall 2,602 patients were analyzed to be potential candidates to participate the study. 465 patients (17.9%) died before follow-up visit. In addition, 1,443 patients (55.5%) were not available or did not react to the invitations due to unknown reasons. 326 patients (12.5%) refused to participate the study. 229 patients (8.8%) were excluded due to minor TBI. Finally, 135 traumatized patients fulfilling the inclusion criteria were included for this study (5.3%). 27 children suffered from moderate to severe TBI in the pre-school group, 32 children in the school group and 76 patients in the adult group. Adult patients were significantly more often of male gender compared to children (Table 1). Mean age at the time of injury was 4.0 ± 2.1 years in the pre-school group, 12.1 ± 3.1 years in the school group, and 38.0 ± 13.1 years in the adult group. None of the participants reported life changing events that might have influenced the measured outcome parameters subjectively.

### Injury severity

No differences were found according to the maximum AIS, ISS or the initial GCS between the age groups (Table 2). Focusing on the morphological injury severity measured by CT scan, the pre-school group demonstrated more often minor injuries (diffuse injury II and III) compared to school-aged and adult patients (p = 0.007). In the school group “no visible pathology” in CT scan was diagnosed most frequently (37.5%) while the highest incidence of surgical interventions was found in adult patients (50%) compared to both children groups (p = 0.007) (Table 2).

**Table 2:** Demographic results and injury severity according to the study groups

	**Pre-school**	**School**	**Adult**	**p-value**
Number of patients (n)	27	32	76	-
Age at time of injury (years)	4.0 ± 2.1	12.1 ± 3.1	38.0 ± 13.1	<0.001
[Min.–Max.]	[0–7]	[8–17]	[19–63]	
Age at follow-up (years)	17.6 ± 5.5	27.6 ± 6.7	51.9 ± 13.1	<0.001
Time since injury (years)	13.7 ± 4.4	14.7 ± 7.0	13.9 ± 2.8	0.482
Gender distribution (♂)	16 (59.3%)	17 (53.1%)	61 (80.3%)	0.008
Initiale GCS	9.1 ± 5.4	6.2 ± 5.1	8.1 ± 5.6	0.119
Maximum AIS head	3.6 ± 1.1	3.8 ± 1.5	4.0 ± 0.9	0.229
ISS	14.7 ± 6.3	16.1 ± 9.0	18.7 ± 7.9	0.054
TCDB classification				
I	7 (25.9%)	12 (37.5%)	5 (6.6%)	0.007
II	12 (44.4%)	9 (28.1%)	30 (39.5%)	
III	2 (7.4%)	1 (3.1%)	2 (2.6%)	
IV	0	0	1 (1.3%)	
V	6 (22.2%)	10 (31.2%)	38 (50.0%)	
VI	0	0	0	

### Physical and psychological long-term outcome

Evaluating the physical long-term outcome, best results according to the GOS score were found in the pre-school group while worst results were measured in adult patients (Table 3). No outcome differences could be observed between the school and the adult group (post hoc p = 0.755). The physical SF-12 and the Barthel score revealed no physical outcome differences between the study groups. Highest scores, however, were measured in the pre-school group (Table 3).

**Table 3:** Physical and psychological long-term outcome after TBI between the study groups

**Physical outcome**	**Pre-school**	**School**	**Adult**	**p-value**
GOS	5.0 ± 0.2	4.5 ± 0.8	4.6 ± 0.6	0.009
SF-12 physical (PCS)	44.6 ± 4.0	43.6 ± 4.3	41.9 ± 5.8	0.060
Barthel score	99.4 ± 2.1	92.3 ± 24.7	94.5 ± 19.3	0.337
**Psychological outcome**				
SF-12 mental (MCS)	56.5 ± 6.6	52.7 ± 7.9	53.3 ± 9.4	0.215
HADS anxiety	1.7 ± 2.4	4.3 ± 3.4	4.1 ± 4.2	0.010
HADS depression	0.6 ± 1.4	1.6 ± 2.7	2.5 ± 3.6	0.026
IES score	1.8 ± 5.4	6.8 ± 12.3	6.2 ± 12.3	0.164

Emphasizing on the long-term psychological outcome (Table 3), no differences between the study groups were found according to the IES score and the psychological SF-12. Regarding the mean HADS scores, an increasing incidence of anxiety and depression was associated with increasing age. Comparable results for anxiety (post hoc p = 0.970) and depression (post hoc p = 0.405) were found between the school group and the adult group. Dividing the HADS anxiety and depression scales to cases, borderline cases and non-cases, no differences could be analyzed between the study groups neither with respect to anxiety (χ^2^ = 7.060, p = 0.315) nor to depression (χ^2^ = 5.086, p = 0.533) (Table 4).

**Table 4:** HADS categories referring to the study groups

**HADS category anxiety**	**Pre-school**	**School**	**Adult**
Cases	0	1 (3.1%)	6 (7.9%)
Borderline	1 (3.7%)	5 (15.6%)	6 (7.9%)
Non-cases	26 (96.3%)	25 (78.1%)	61 (80.3%)
Not evaluable	0	1 (3.1%)	3 (3.9%)
**HADS category depression**			
Cases	0	0	3 (3.9%)
Borderline	0	2 (6.5%)	6 (7.9%)
Non-cases	26 (96.3%)	28 (90.3%)	63 (82.9%)
Not evaluable	1 (3.7%)	1 (3.2%)	4 (5.3%)

### Age at time of injury and injury severity as outcome predictor

We found no significant correlation between the age at time of injury and the measured physical as well as psychological outcome parameters (Table 5). However, physical outcome measured by GOS was strongly associated with the injury severity according to the GCS, AIS head, ISS and TCDB classification. A lower initially raised GCS score was associated with a lower GOS score. On the other side, increased AIS, ISS and TCDB scores were followed by decreased GOS. The raised long-term outcome parameters with respect to Barthel score and SF-12 were neither associated with the injury severity nor the age at time of injury.

**Table 5:** Correlation of age at time of injury, injury severity and long-term outcome

		**GOS**	**Barthel score**	**SF 12 physical (PCS)**	**SF-12 mental (MCS)**	**IES score**	**HADS anxiety**	**HADS depression**
**Age at time of injury**	Correlation coefficient	-0.046	0.005	-0.113	-0.070	0.070	0.071	0.152
	p-value	0.593	0.958	0.203	0.430	0.417	0.420	0.086
**Initial GCS**	Correlation coefficient	0.284	0.109	-0.062	0.067	-0.009	-0.079	-0.082
	p-value	0.001	0.217	0.497	0.462	0.918	0.379	0.370
**Max. AIS head**	Correlation coefficient	-0.288	-0.149	-0.104	-0.094	-0.034	0.126	0.076
	p-value	0.001	0.087	0.245	0.296	0.700	0.156	0.398
**ISS**	Correlation coefficient	-0.285	-0.168	-0.079	-0.084	-0.005	0.144	0.091
	p-value	0.001	0.053	0.379	0.348	0.957	0.105	0.313
**TCDB**	Correlation coefficient	-0.240	-0.124	-0.129	-0.006	0.014	-0.043	0.077
	p-value	0.005	0.153	0.148	0.949	0.845	0.627	0.386

Focusing on the psychological parameters, no correlation was found referring to the measured injury severity scores (Table 5).

## Discussion

Several factors are suspected to influence long-term outcome after pediatric TBI [10]. Beside the injury severity as critical predictor [10], age at time of injury has been suggested to have a significant impact on functional and cognitive recovery [3,5,10,11]. In order to reveal general physical and psychological outcome differences after moderate to severe TBI between miscellaneous age groups we found the following results:

– Physical and psychological long-term outcome was not associated with the age at time of injury but with the injury severity.

– General outcome recovery measured by GOS was demonstrated to be best in the pre-school group.

– SF-12 and Barthel scores were comparable between pediatric and adult patients.

– According to several psychological outcome scores an increasing incidence of anxiety and depression was found to be associated with increasing age.

According to previous studies, our findings confirmed the significant impact of injury severity on long-term outcome: Comparable to our results with a significant correlation between the injury severity and GOS outcome, Catroppa et al. found similar associations analyzing functional outcome after pediatric TBI. In their study, injury severity measured by GCS was revealed as a predictor for behavioral outcome as well as educational performance 5 years after trauma [9]. Also in dependence of TBI severity, Anderson et al. reported that pre-school children suffered from depression of intellectual abilities 10 years after trauma compared to a normative population [5]. Most significant effects of a high injury severity (measured by GCS and white matter volume) were found on adaptive and social abilities [5], with the GCS on admission correlated with IQ performance [5]. However, perceptions out of this study might be restricted due to the fact that the injury severity was only measured by GCS. In this context, a weak reliability of the GCS towards outcome in very young children has already been suggested [38]. In the presented study, we analyzed outcome even children at very young age. Accordingly, Crowe et al. were not able to find a predictive value of the GCS for posttraumatic intellectual, behavioral and social performance in children with moderate and severe TBI [11]. We therefore extended the injury severity measurement in our study by additional CT diagnostics, which is a central part of the decision making process in pediatric traumatic brain injuries because of the quick detection of surgically relevant lesions [39]. CT diagnostic was found inferior for the detection and assessment of traumatic lesions compared to MRI, it might be assumed that CT-based outcome perceptions might be limited compared to MRI [40]. However, CT has been recommended as an essential part of the acute traumatic brain injury diagnostic protocol to assess the need for neurosurgical intervention [41].

With respect to long-term perceptions only the GOS correlated with an increased injury severity in the presented study. In this context, a previous study showed that even children with most severe brain injuries, who enter rehabilitation completely dependent for all daily activities, have the potential to make significant gains in functioning by discharge and in the following few months [42]. Therefore, it might be assumed that it is difficult to find associations between scores like the Barthel Index and SF-12 measurements and the initially evaluated injury severity after a follow-up time of 10 years after trauma.

With regards to psychological outcome, we found no association with the severity of TBI. Accordingly, Max et al. reported that posttraumatic psychiatric disorders occurred significantly more often after pediatric TBI but were not associated to the injury severity or age at time of injury [43]. Also in accordance with our results, Greenspan et al. were not able to correlate TBI severity with the IES score [37]. The authors hypothesized that psychological symptoms might emerge independently from TBI deficits. In contrast, Hawley et al. compared HADS scores of different TBI severity groups and found a higher incidence of anxiety and depression in case of moderate to severe TBI compared to a healthy control population [33]. As neither a correlation nor regression analysis was performed in the study of Hawley et al., comparability to our results might be limited. As we did not find any correlation to the injury severity or the age at time of injury one might suggest that family support and further social circumstances during the follow-up time of 10 years could have lead to these results. In this context, Yeates et al. have reported that family environment moderates the psychosocial outcomes of TBI in young children, but the influence might wane with time among children with severe TBI [15]. However, as we did not raise any parameters evaluating the influence of the family environment towards outcome conclusions cannot be drawn focusing this aspect.

As no associations between patient’s age and psychological as well as general functional long-term outcome 10 years after trauma were found in our study, we believe that current clinical results [3,5,10,11] of deteriorated recovery for the immature brain have to be questioned for the long-term. In this context it has to be mentioned that follow-up periods in previous studies of Anderson et al. and Crowe et al. were significantly shorter compared to the presented study. Anderson et al. reevaluated the included patients after 30 months [10] and five years [3], while Crowe et al. reexamined their patients after 40 months [11]. It might therefore be assumed that recovery is time-dependent even several years after trauma. Accordingly in a recent study by Anderson et al. [5] with a follow-up period of 10 years no significant impact of age at time of injury on cognitive function was detected, which is similar to the results of our study. Furthermore, Catroppa et al. were recently also not able to verify the age at time of injury as an independent predictor for long-term outcome 10 years after trauma [12]. In brief summary the authors constituted that age at injury effects might have varied across the study sample and that these effects might have been nonlinear in nature [5]. In addition, one might argue this effect by the prolonged follow-up period of 10 years which could have influenced physical and psychological outcome compared to shorter follow-up periods [42]. It might also be assumed, that our results are caused by the study design. In this context the aforementioned studies compared traumatized children with healthy, uninjured controlled populations [3,5,9,11,12]. To the best of our knowledge the presented study is uniquely demonstrating a direct comparison of traumatized patients of different age groups and comparable trauma impact with a follow-up period of at least 10 years.

Nevertheless, the presented study has several limitations which should be considered when interpreting the demonstrated results. Due to the follow-up period of at least 10 years and its retrospective design, many critical events might have occurred in a persons’ life potentially affecting outcome. Although the participating patients have been asked for life-changing events between the TBI and follow-up, this aspect has to be considered as a potential limitation when interpreting the results. Especially pre-existing psychological and behavioural problems might be missed by this study, because none of the traumatized patients was assessed by specific psychological scores on admission when treated for TBI. We excluded patients with mental handicaps previous to TBI, but minor problems were potentially missed by this study. As these problems might interfere with the presented outcome results, this aspect should be taken into account when interpreting the presented results. Furthermore, the length of follow-up and data collection at a single center might be a limitation and it is likely that the presented findings cannot reflect the advances made in acute care as well as rehabilitation during the last decades. Additionally, one might be aware of a potential selection bias which is a known limiting aspect of long-term outcome studies especially when arguing on the finally included patients. Participants were separated to pre-school aged, school aged, and adult patients in the present study. In this respect, the school aged group represented a wide range of age with the final neural growth in this age group. Outcome differences within this group might have been masked due to the study design. Although there were no differences in injury severity, there were differences in TCDB classification, with significant more in the adult group having a more severe rating. This may have biased the findings, and could potentially be one reason for the differences found by this study.

In the present study outcome measurements were mainly questionnaire based, and no cognitive measures were employed. These aspects have to be taken into account when interpreting the presented results.

## Conclusions

The presented long-term outcome perceptions after moderate to severe TBI question current clinical and experimental results of deteriorated recovery for the immature brain. Pediatric TBI during a potentially vulnerable phase referring to cognitive skill maturation at pre-school age seemed not to impair functional and psychological long-term outcome compared to elder children or adults. Consequently, sustained TBI deficits seemed not to reduce the child’s ability to manage or minimize the suffered impairment. The association of injury severity and outcome deficits diminished referring to long-term outcome distinguishing the relevance of rehabilitation and family support in the long term.

## Abbreviations

AIS: Abbreviated injury scale; ANOVA: Analysis of variance; CT: Computed tomography; GCS: Glasgow coma scale; GOS: Glasgow outcome score; HADS: Hospital anxiety and depression scale; IES: Impact of event scale; ISS: Injury severity score; MCS: Mental component summary scale; PCS: Physical component summary scale; SF-12: Short form 12; TCDB: Traumatic coma databank; TBI: Traumatic brain injury.

## Competing interests

The authors declare that they have no competing interests.

## Authors’ contributions

HA conceived this study designing the trial, provided statistical advice on study design, analyzed the data and drafted the manuscript. He takes responsibility for the article as a whole. ED, JU raised and analyzed the data, drafted the manuscript, and approved the final manuscript as submitted. CP, CK conceived the study, obtained research funding and designed the trial, raised the data, and approved the final manuscript as submitted. OG and RL conceived this study designing the trial, provided statistical advice on study design, and approved the final manuscript as submitted. MF analyzed the data, reviewed the manuscript and approved the final manuscript as submitted. CZ, CK and FH conceived the study, obtained research funding and designed the trial, raised the data, reviewed the manuscript and approved the final manuscript as submitted. All authors read and approved the final manuscript.
